# Association of Breastfeeding Duration, Nonnutritive Sucking Habits, and Malocclusion

**DOI:** 10.5005/jp-journals-10005-1477

**Published:** 2017-02-01

**Authors:** Marina G Roscoe, Sara V da Silva Bonifacio, Teddy B da Silva, Joao MS Pingueiro, Maurilo M Lemos, Murilo FN Feres

**Affiliations:** 1 Assistant Professor, Dental Research Division, Department of Orthodontics Guarulhos University, Sao Paulo, Brazil; 2 Undergraduate Student, Department of Pediatric Dentistry and Orthodontics, Sao Francisco University, Sao Paulo, Brazil; 3 Undergraduate Student, Department of Pediatric Dentistry and Orthodontics, Sao Francisco University, Sao Paulo, Brazil; 4 Postgraduate Student, Dental Research Division, Department of Orthodontics Guarulhos University, Sao Paulo, Brazil; 5 Assistant Professor, Dental Research Division, Department of Orthodontics Guarulhos University, Sao Paulo, Brazil; 6 Assistant Professor, Dental Research Division, Department of Orthodontics Guarulhos University, Sao Paulo, Brazil

**Keywords:** Breastfeeding, Malocclusion, Nonnutritive sucking habits, Orthodontics.

## Abstract

**Aim:**

This study aimed to investigate the associations between breastfeeding, nonnutritive sucking habits (NNSHs), and malocclusion in deciduous, mixed, and permanent dentition.

**Materials and methods:**

A sample of 50 children between 3 and 12 years, enrolled in a pediatric dentistry dental care program, underwent orthodontic examination for detection of occlusal patterns and malocclusion. In addition, data regarding breastfeeding duration and NNSH acquisition were obtained from standardized questionnaires responded by the children’s parents or legal guardians.

**Results:**

Regardless of a long period of breastfeeding, a high incidence of NNSH in the evaluated sample was observed. Nevertheless, the presence of NNSHs was not significantly associated with malocclusion.

**Conclusion:**

The findings could not indicate a statistically significant association between breastfeeding duration, acquisition of NNSHs, and malocclusion. Longitudinal studies with larger samples are still needed to better support clinical decisions.

**How to cite this article:** Roscoe MG, da Silva Bonifacio SV, da Silva TB, Pingueiro JMS, Lemos MM, Feres MFN. Association of Breastfeeding Duration, Nonnutritive Sucking Habits, and Malocclusion. Int J Clin Pediatr Dent 2018;11(1):18-22.

## INTRODUCTION

Nonnutritive sucking habits, such as the use of pacifiers and digit-sucking, are common in babies and young children.^1^ It is suggested that the maintenance of these habits while the permanent dentition is becoming established might contribute to the development of malocclusions.^1^ In order to prevent and intercept occlusal disturbances, the investigation of factors associated to the early occurrence of malocclusions becomes very important, especially when planning public health policies.^2^

Considering the psychological positive effects that breastfeeding provides, the World Health Organization recommends it to be exclusive for at least 6 months.^3,4^ Breastfeeding has been considered as a determining factor for proper craniofacial development, because it promotes intense exercise of the orofacial muscles,^5^ favorable stimulation of mandibular sagittal growth, correction of the intermaxillary relationship, in addition to protection against malocclusion, especially if prolonged, since it prevents the acquisition of NNSH.^6-11^

However, there are still controversies regarding the period of time breastfeeding might be present so that it can effectively prevent NNSHs to take place.^12,13^ Therefore, this study aimed to investigate the associations between breastfeeding duration, acquisition of NNSHs (pacifier-sucking, finger-sucking, and bottle-feeding), and malocclusion in the deciduous, mixed, and permanent dentition.

## MATERIALS AND METHODS

The research project was submitted and approved by the Ethics Committee on Research Involving Humans (protocol no 237693). The sample of this retrospective study consisted of 50 children of both genders between 3 and 12 years, which were enrolled in a Pediatric Dental Care Program of the Sao Francisco University (Braganca Paulista, Sao Paulo, Brazil). Children that were resistant to oral examination, presented with malformations or craniofacial syndromes, and those who had undergone orthodontic treatment were excluded from the study. Of the total of 112 children, 52 (46.4%) did not attend the scheduled days of the research, 8 (7.1%) had undergone orthodontic treatment, and 2 (1.7%) demonstrated resistance to oral examination. The final sample was thus composed by 50 children.

Data regarding the duration of breastfeeding and the acquisition of NNSHs during early childhood were obtained from questionnaires. A single independent and trained examiner conducted the interviews with the parents or legal guardians, by using a structured questionnaire concerning pacifier, digit-sucking, bottle-, and breastfeeding. Simultaneously, another examiner performed the orthodontic clinical examinations. Intraoral examinations were performed by using artificial light, intraoral mirror, periodontal probe, and compass.

Overjet was obtained by measuring the distance between the incisal edge of the most prominent upper incisor and the buccal surface of the corresponding lower incisor with the use of a periodontal probe, positioned parallel to the occlusal plane. Overjet was categorized as excessive (>3 mm), normal (between 0 and 3 mm), or negative, when an anterior crossbite was observed. Overbite was obtained by measuring the vertical distance between the upper and lower central incisor edges. Subjects were categorized as having deep (>3 mm), normal overbite (between 0 and 3 mm), or anterior open-bite, when there was no horizontal overlap between the upper and lower incisors. Crossbite was considered present when the buccal cusps of the mandibular molars were buccally displaced in relation to the buccal cusps of the upper molars. Patients were classified as class I, II, and III, according to the Angle classification (molar relationship) and in normal, distal, and mesial relationship, according to the upper and lower canine sagittal position. The molar relationship was considered: (a) Class I, when the mesiobuccal cusp of the maxillary first molar was aligned with the buccal groove of the mandibular first molar; (b) class II, when the buccal groove of the mandibular first molar was distally positioned in relation to the mesiobuccal cusp of the maxillary first molar; and (c) class III, when the buccal groove of the mandibular first molar was mesially positioned to the in relation mesiobuccal cusp of the maxillary first molar when the teeth are in occlusion. The canine relationship was considered: (a) Normal, when the distal slope of the lower canine occluded with the mesial slope of the upper canine; (b) distal, when the mesial slope of the lower canine occluded with the distal slope of the upper canine; and (c) mesial, when the lower canine was further mesially positioned than the upper canine.

Categorical variables were described with absolute and relative frequencies (percentage). Descriptive analysis of the quantitative variables included mean and standard deviation calculations. Inferential statistics were performed by association tests between categorical and numerical variables and correlation tests between quantitative variables. The significance level for statistical tests was 5% (a < 0.05).

## RESULTS

The final sample consisted of 29 boys (58.0%) and 21 girls (42.0%), and mean age was 8.2 years [standard deviation (SD) = 2.0]. According to the results obtained by interviews, 49 children (98%) were breastfed, during 10.8 months (SD = 9.9), on average. Bottle-feeding was reportedly present in 44 children (88.0%) during 3.3 years (SD = 1.8), on average. Pacifier was used by 27 children (54.0%) during 3.9 years (SD = 2.2), on average. Digit-sucking was reported for only 5 children (10%). Table 1 indicates that most children were in the mixed dentition (41, 82.0%) and presented overbite and overjet of up to 3 mm, which was considered normal (38, 80.9%; 32, 65.3% respectively) (Table 1). Only 8 children presented posterior crossbite (16.0%). Concerning sagittal relationship, most children presented Class I molar and normal canine relationship (23, 57.5%; 28, 63.6% respectively).

Table 2 indicates that children who bottle-feed were breastfed for significantly shorter periods of time in relation to those that did not bottle-feed. However, this trend was not identified for pacifier, neither for digit-sucking habits. Moreover, association between bottle-feeding and malocclusion of any kind was not observed, since no statistical difference was detected when children with and without history of bottle-feeding were compared (Table 3).

The use of pacifier was not associated with overjet, posterior crossbite, canine, or molar relationship. Even though the incidence of open bite was higher among the children that used pacifier, this difference was not statistically significant (Table 4). Due to the low frequency of digit-sucking habit (5, 10.0%), its association with occlusal features was not investigated.

**Table 1:** **Table 1:** Occlusal features concerning dentition, overbite, overjet, and Angle’s classification

*Dentition (n/%)*							
*Deciduous*		*Mixed*		*Permanent*		*Total*	
6/12		41/82		3/6		50/100	
*Overbite (n/%)*							
Normal		Overbite		Open bite		Total	
38/80.9		5/10.6		4/8.5		47/100*	
*Overjet (n/%)*							
Normal		Overjet		Crossbite		Total	
32/65.3		16/32.7		1/2.0		49/100*	
*Angle’s classification (n/%)*							
Class I		Class II		Class III		Total	
23/57.5		17/42.5		0/0		40/100*	
*Canine relationship (n/%)*							
Normal		Distal		Mesial		Total	
28/63.6		16/36.4		0/0		44/100*	

*The total number of patients evaluated for each parameter differed due to the impossibility of classification (tooth absence)

**Table 2:** **Table 2:** Relationship between acquisition of NNSHs and breastfeeding duration

				*Breastfeeding duration (months)*							
*NNSHs*				*n/%*		*Mean*		*SD*		*p-value*	
Bottle-feeding		Yes		44/88		9.3		9.3		0.004	
		No		6/12		21.3		7.9			
Pacifier		Yes		27/54		8.9		10.8		0.148	
		No		23/46		13.0		8.3			
Digit-sucking		Yes		5/10		9.3		6.2		0.748	
		No		45/90		10.9		10.2			

## DISCUSSION

Given the lack of publicly funded dental treatment programs in developing countries, it becomes increasingly important to identify etiological factors that can be addressed through preventive and interceptive orthodontics.^14^ For instance, insufficient breastfeeding is correlated to the underdevelopment of the masticatory complex, the onset of mouth breathing, tongue thrusting, introduction of other habits, and therefore, to malocclusion.^15^ According to the results obtained in the present study, 98% of the sample evaluated in this study was breastfed for more than 6 months (10.8 months, on average).

**Table 3:** **Table 3:** Relationship between bottle-feeding habit and occlusal features

				*Bottle feeding*													
				*Yes*				*No*				*Total*				*p-value*	
Overbite		Normal		33		80.5%		5		83.3%		38		80.9%		0.743	
		Overbite		4		9.8%		1		16.7%		5		10.6%			
		Open bite		4		9.8%		-		-		4		8.5%			
Overjet		Normal		27		62.8%		5		83.3%		32		65.3%		0.691	
		Overjet		15		34.9%		1		16.7%		16		32.7%			
		Crossbite		1		2.3%		-		-		1		2.0%			
Posterior crossbite		Yes		7		15.9%		1		16.7%		8		16.0%		>0.999	
		No		37		84.1%		5		83.3%		42		84.0%			
Angle’s classification		Class I		18		52.9%		5		83.3%		23		57.5%		0.216	
		Class II		16		47.1%		1		16.7%		17		42.5%			
		Class III		-		-		-		-		-		-			
Canine relationship		Normal		24		60.0%		4		100.0%		28		63.6%		0.280	
		Distal		16		40.0%		-		-		16		36.4%			
		Mesial		-		-		-		-		-		-			

**Table 4:** **Table 4:** Relationship between pacifier sucking habit and occlusal patterns evaluated

*Pacifier use*																	
				*Yes*				*No*				*Total*				*p-value*	
Overbite		Normal		17		68.0%		21		95.5%		38		80.9%		0.061^a^	
		Overbite		4		16.0%		1		4.5%		5		10.6%			
		Open bite		4		16.0%		-		-		4		8.5%			
Overjet		Normal		16		61.5%		16		69.6%		32		65.3%		0.450^a^	
		Overjet		10		38.5%		6		26.1%		16		32.7%			
		Crossbite		-		-		1		4.3%		1		2.0%			
Posterior crossbite		Yes		5		18.5%		3		13.0%		8		16.0%		0.711^a^	
		No		22		81.5%		20		87.0%		42		84.0%			
Angle’s classification		Class I		11		55.0%		12		60.0%		23		57.5%		0.749^b^	
		Class II		9		45.0%		8		40.0%		17		42.5%			
		Class III		-		-		-		-		-		-			
Canine relationship		Normal		13		54.2%		15		75.0%		28		63.6%		0.153^b^	
		Distal		11		45.8%		5		25.0%		16		36.4%			
		Mesial		_-_		_-_		_-_		_-_		_-_		_-_			

^a^Fisher’s exact test, ^b^Chi-square test

Most children presented normal overbite and overjet (80.9 and 65.3% respectively), Class I and normal canine relationship (57.5 and 63.6% respectively), and only 8 children presented posterior crossbite (16.0%). The main malocclusion condition found was excessive overjet, present in approximately one-third of the sample (32.7%). The literature presents high frequencies of malocclusion among children, varying from 60,^15,16^ 70^17,18^ up to 73%.^19^ The fact that breastfeeding was frequently reported might have contributed to the low percentage of malocclusion identified in the present study.

Even with high rates and long periods of breastfeeding (98% of the total sample breastfeed for 10.8 months on average), NNSHs were present in most of the children. There are still controversies regarding the interaction between breastfeeding with deleterious oral habits. Some studies indicate that breastfeeding can prevent the occurrence of sucking habits.^20,21^ Our results were not in accordance with literature, since bottle-feeding and pacifier-sucking were frequently reported (88 and 54% respectively). Digit-sucking was reported to be present in only five children (10%). For nutritional purposes, children who were breastfed for shorter periods of time introduced bottle-feeding, more frequently, when compared with children who were breastfed for longer periods.

Regarding the interaction between NNSHs and maloc-clusions, it was observed that pacifier-sucking children presented, on average, reduced overbite. Still, this difference was not statistically significant. Regarding the others occlusal parameters evaluated (overjet, posterior cross-bite, molar, and canine relationship), no difference was detected comparing the groups. These results support the evidence that genetic factors might play a major role in the development of the facial pattern.^7,22,23^

There are evidences supporting that deleterious oral habits might negatively affect dental arch development.^24^ For instance, prolonged NNSHs, such as pacifier- and digit-sucking are generally associated with reduced overbite, increased overjet, and posterior crossbite.^13,25-28^ Still, malocclusions could have a physiological resolution if the deleterious habit is interrupted until 4 years of age because until this age there is a potential for self-correction of malocclusion,^29^ which might have occurred in some patients of the present sample.

The results of the present study do not support a significant relationship between breastfeeding duration, acquisition of NNSHs, and development of malocclusion. Still, the literature regarding the effects of breastfeeding on occlusion is inconclusive and, therefore, it is suggested to conduct longitudinal studies with larger samples to detect more associations between the presence of NNSHs and the signs of malocclusion to better support the clinical decisions.

## CONCLUSION

The present findings could not indicate a statistically significant association between breastfeeding duration, acquisition of NNSHs, and malocclusion.

## CLINICAL SIGNIFICANCE

Long breastfeeding duration cannot be regarded as a protective factor against the acquisition of NNSHs, since no statistically significant association was found between breastfeeding duration, acquisition of NNSHs, and the occlusal pattern.
